# Proteomic Analysis of Human Esophageal Cancer Using Tandem Mass Tag Quantifications

**DOI:** 10.1155/2020/5849323

**Published:** 2020-08-07

**Authors:** Suofeng Sun, Huijuan Zhang, Yu Wang, Jing Gao, Shen Zhou, Yuan Li, Shuangyin Han, Xiuling Li, Jian Li

**Affiliations:** ^1^Department of Gastroenterology, Henan Provincial People's Hospital and the People's Hospital of Zhengzhou University, Zhengzhou, China 450000; ^2^Medical Care Department, First Affiliated Hospital of Hainan Medical College, Haikou, Hainan, China 570102; ^3^Xinxiang Medical University, Zhengzhou, China 450000; ^4^Department of Traditional Chinese Medicine, The Third Affiliated Hospital Affiliated of Henan University of Traditional Chinese Medicine, Zhengzhou, Henan, China 450000

## Abstract

Esophageal cancer (EC) is a type of extremely aggressive gastrointestinal cancer with high incidences in China and other Asian countries. EC does not have specific symptoms and is relatively easy to metastasize, which makes it difficult in early diagnosis. Thus, novel noninvasive diagnostic method is urgently needed in clinical practice. In this study, mass spectrometry with tandem mass tags and differential protein analysis were applied for identifying esophageal cancer-related proteins. The identified proteins were annotated based on their enrichment in Gene Ontology (GO) terms and Kyoto Encyclopedia of Genes and Genomes (KEGG) pathways. In addition, hierarchical clustering was applied based on differentially expressed proteins. As a result, a total of 5131 quantifiable proteins were identified from our liquid chromatography-tandem mass spectrometry with tandem mass tags (LC-MS/MS-TMT) method with 63 upregulated and 97 downregulated differential proteins between esophageal cancer and controlled normal samples. The differentially expressed proteins were highly enriched in GO terms associated with mitochondrial dissemble and apoptosis, and blood vessel regulation, and the upregulated differentially expressed proteins in EC samples were significantly enriched in major histocompatibility complex MHC-class I/II pathway of immune system. The functional clustering analysis revealed potential protein-protein interactions among tetraspanin, myosin, and S-100. In summary, our study provided a practical technological procedure of proteomic analysis for discovering novel biomarkers of a specific cancer type.

## 1. Introduction

Esophageal cancer (EC) processes a significant health risk due to increasing incidence and poor prognosis [1]. As an extremely aggressive neoplasm, approximately 45,000 people are diagnosed with EC each year, while the overall 5-year survival rate is less than 10%. Although chemotherapy and adjuvant chemotherapy are widely used in the treatment of esophageal cancer, the prognosis remains poor due to high possibility of tumor relapse or distant metastasis. Beginning in the mucosa of the esophagus and spreading through a deeper tissue layer, such as the submucosa, muscular layer, and serosa, esophageal cancer cells are able to metastatic progress through lymphatic and homogametic vessels. The most common pathologically histological types are squamous cell and adenocarcinoma, which usually occur at the lower (distal) part of the esophagus [2–4]. Endoscopy typically shows a tumor mass at the distal or gastroesophageal junction. One hypothesis of the esophageal cancer model is the squamous epithelium undergoing chronic inflammatory changes, leading the changes in the cell structure and shape and in situ malignant changes as well [5–7].

The risk factors of esophageal carcinoma include history of symptomatic gastroesophageal reflux disease, tobacco use, and alcohol intake. Barium esophagography is widely used as the initial assessment in patients with symptoms of esophageal carcinoma [8, 9]; however, the confirmed diagnosis based on biopsy tissue required an invasive physical procedure [10, 11]. Dysphagia with weight loss is the only symptom of esophageal cancer in early stage; esophageal cancers are commonly diagnosed at advanced stages, highlighting the need for improved detection and prediction methods with novel biomarkers. Furthermore, esophageal cancer in advanced stage was insensitive to chemoradiotherapy making therapeutic even challenging; diagnosis based on biomarkers in the early stage will benefit the prognosis and overall survival rate of EC patients. Previous studies have demonstrated that many proteins play an important role in tumor development and their abnormal expressions are associated with cancer cell proliferation and migration, such ATP-binding cassette protein E1 [12] and loss of PAR4 gene expression [13, 14].

Proteomics has been identified as a powerful approach for novel disease biomarkers discovery [15–17], and mass spectrometry with tandem mass tags (TMT) for proteomics profiling has been reported in various cancer types. Zhang et al. reported plasma exosomes from an ovarian cancer patient from detection by LC-MS/MS with TMT containing tumor-specific proteins relevant to tumorigenesis and metastasis, while Hou et al. developed a drug anti−HCC efficacy method for hepatocellular carcinoma [18–20].

In this study, we applied the tandem mass tag- (TMT-) based quantitative proteomic technique for esophageal cancer proteomics profiling annotation biological meaning and comparing protein-protein interaction. Our study identified potential biomarker for early diagnosis to discover relative effective therapy in the future clinical practices.

## 2. Materials and Methods

### 2.1. Protein Extraction and TMT/iTRAQ Labeling

Tissue samples of three esophageal cancer patients were collected and sonicated for 5 min (30 s on and 30 s off) by a high-intensity ultrasonic processor (Ningbo Scientz Biotechnology, China) in lysis buffer with protease inhibitor cocktail. The protein solution was centrifuged for 15 min and reduced at 56°C with dithiothreitol, diluted with 100 mM TEAB, and digested with trypsin (mass spec grade) for 5 hours. Strata-X C18 SPE column (Phenomenex, CA) was applied for desalting, and the samples were vacuum-dried for further TMT labeling. TMT/iTRAQ labeling was processed according to the TMT/iTRAQ kit with 2 hours at RT, followed with desalting and dried with vacuum centrifugation.

### 2.2. Fractionation of Tryptic Peptides and LC-MS/MS Analysis

Thermo BetaSil C18 column was applied for trypsin-digested peptides, separation used with high-pH reversed-phase HPLC. Solvent B containing 0.1% formic acid in 98% acetonitrile has increasing gradient from 6% to 23% and further to 80% on EASY-nLC 1000 UPLC system with 400 nL/min CFR. The peptides were then analyzed by tandem mass spectrometry in Q Exactive™ Plus (Thermo Fisher Scientific, MA) with full-scan setting of m/z from 300 to 1800 and resolution of 70000 for Orbitrap. NCE setting of 28 was selected for intact fraction detection and selected for further 20 MS scans with 10 s dynamic exclusion.

### 2.3. Proteomics Analysis and Annotations

The MaxQuant search engine was used for LC-MS/MS-TMT result analysis, and tandem mass spectra were searched against human UniProt database concatenated with reverse decoy database. The mass tolerance was set as follows: 0.02 Da for fragment ions and 5 ppm of main search for precursor ions. Fixed modification and variable modifications were specified with carbamidomethyl on Cys and oxidation on Met, respectively. GO annotation was applied for differentially expressed proteins with a biological process, cellular compartment, and molecular function, and enrichment was tested with two-tailed Fisher's exact. Each category protein was also searched in InterPro database for biological function analysis. Kyoto Encyclopedia of Genes and Genomes (KEGG) database was applied for enriched pathway identification. All annotation analyses with a corrected *p* value < 0.05 were considered significant.

### 2.4. Enrichment-Based Clustering and Protein-Protein Interaction Network

We used woLF PSORT a subcellular localization predication soft to predict subcellular localization. WoLF PSORT is an updated version of PSORT/PSORT II for the prediction of eukaryotic sequences. Special for protokaryon species, subcellular localization prediction soft CELLO was used. We applied hierarchical clustering based on differential expressed protein functional classification and collated all the categories obtained after enrichment along with their -log10 (*p* value) and then normalized with *z*-score for hierarchical clustering in Genesis. Cluster membership was visualized by “heatmap.2” function from the “gplots” R-package. STRING database (version 10.1) was used for the prediction of protein-protein interactions with differentially expressed proteins, and external candidates were excluded. The confidence score was calculated for the connection evaluation of protein-protein interaction, and 0.7 was set as the cut-off for PP interaction.

## 3. Results

### 3.1. Identification of Differentially Expressed Proteins by LC-MS/MS-TMT

**Figure 1:**
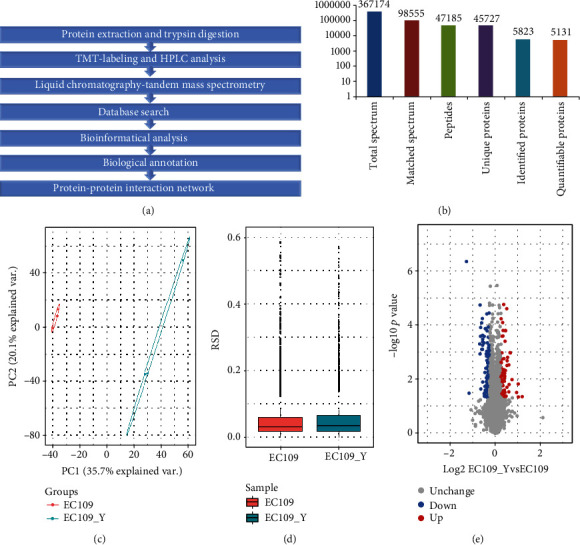
Overall design and protein identification. (a) Overall study design and analysis process. (b) Protein identification from 367174 secondary spectrograms. (c) PCA analysis between EC109 and EC109_T. (d) Boxplot of RSD distribution for protein quantification evaluation between replicates. (e) Differentially expressed protein analysis between EC tumor and control samples.

Extracted proteins from esophageal cancer samples were quantified by LC/LC-MS with TMT as shown in Figure 1(a). A total of 367174 secondary fingerprint spectra were obtained through mass spectrometry, and 98555 (26.8%) effective spectrograms obtained were filtered by MaxQuant. 47185 peptides were identified by spectrogram analysis with 96.9% unique peptides. Overall, 5823 proteins were identified with database and 5131 (88.12%) proteins could be quantifiable with comparable information in the control group (Figure 1(b) and Supplementary Table 1). Principal component analysis (PCA) was performed to validate the quantitative results between the biological triplicate samples, and the EC109 group shows better repeatability (Figure 1(c)). Relative standard deviation (RSD) distribution shows that protein quantification evaluation was comparable between replicates and no significant difference in overall protein quantification between EC109 and EC109_T group (Figure 1(d)). The proteins of all samples are quantitatively detected, and differentially expressed protein was defined with *p* value < 0.05 and log2 fold change > 1.2 based on mean expression. We detected 63 upregulated proteins, 97 downregulated proteins between two groups with 57/62/43 upregulated and 60/112/69 downregulated proteins separately (Figure 1(e)).

### 3.2. Functional Analysis of Identified Differentially Expressed Proteins

**Figure 2:**
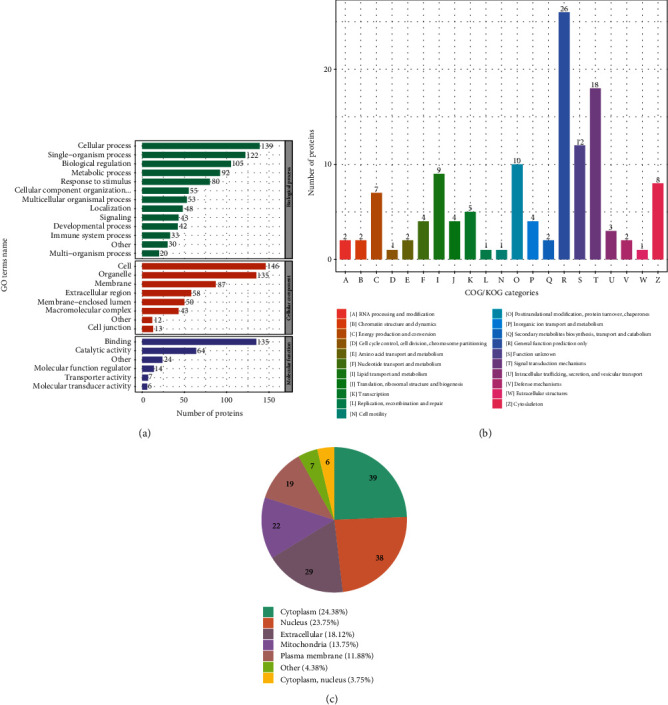
Functional classification analysis of identified differentially expressed proteins. (a) The GO term annotation of quantitive DEP. (b) The distribution of subcellular location of differentially expressed protein. (c) Distribution of COG/KOG function classification.

To further analyze differentially expressed proteins, systematic bioinformatics analysis was applied including functional classification, function enrichment, and enrichment-based clustering analysis. The top three significant biological processes of the differentially expressed protein in this project were (1) cellular process, (2) single-organism process, and (3) biological regulation. For molecular function, the results indicated that many proteins are involved in binding and catalytic activities, meaning those proteins participated in cell-to-cell signaling transduction and facilitated signal pathway regulation. Gene ontology terms of quantified proteins also revealing a response to stimulus and immune system processes are enriched in the biological process, while catalytic activity of differentially expressed protein may be involved in development (Figure 2(a)). Subcellular localization analysis illustrated that cytoplasm accounted for 24.38% in the overall cellular structure, while 13.75% proteins from the mitochondria suggested potential mitochondria-mediated oxidative stress regulation between EC109 and EC109_T groups (Figure 2(b)). We further checked the number of proteins in COG/KOG categories (Clusters of Orthologous Groups of proteins) in all the comparable groups; 28 proteins are associated with signal transduction mechanism and posttranslational modification, revealing that signaling between cells is important in esophageal cancer. Furthermore, 16 other proteins are categorized in lipid transport and metabolism and the protein metabolic pathway, while the metabolic product from lipid metabolism served as long-term energy for cell development and maintaining accurate functions. The energy from lipid metabolism is usually stored in the specific cell and plays a critical role in stress response and survival (Figure 2(c)).

### 3.3. GO Analysis for Esophageal Cancer-Related Proteins

**Figure 3:**
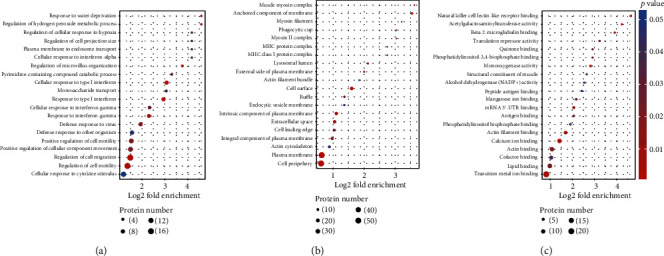
The distribution of differentially expressed proteins between esophageal cancer groups in functional enrichment of (a) biological process, (b) cellular component, (c) molecular function.

Furthermore, we applied enrichment analysis on three GO domains, including biological process cellular component and molecular function of differentially expressed proteins. The biological analysis reveals that most differentially expressed proteins participated in cell regulation and metabolism, such as mitochondrial dissemble and apoptosis, blood vessel regulation as well as downregulation of cell migration and motility. Our results indicate that in an EC patient, the cellular immune response was decreased, cell migration and motility were dysregulated, and the cell circle checkpoint was dysfunctional, which may lead to abnormal cell biological function (Figure 3(a)).

Cellular component analysis shows that MHC protein complex was upregulated, and the anchored component of the membrane, actin cytoskeleton, and intrinsic component of plasma membrane were all downregulated, suggesting that extracellular interaction has been increased and the cytoskeleton remains in unstable status with the risk of collapsing (Figure 3(b)). Maintenance of membrane integrity was found in the molecular function analysis, which is the most important to facilitate extracellular activity such as binding to a specific protein or enzyme functioning (Figure 3(c)).

### 3.4. Protein Structure Domain and KEGG Analysis for Esophageal Cancer-Related Proteins

**Figure 4:**
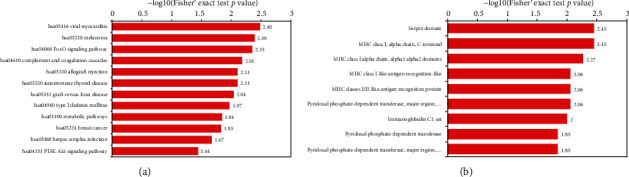
Analysis of regulated protein structure domain (a) and KEGG pathway (b).

Proteins were mostly aggregated in the mucin (specific secretion protein in esophagus) biosynthesis pathway and downregulation of steroid biosynthesis. Furthermore, a recent study reported that mucin synthesis was strongly associated with esophageal cancer progress. Meanwhile, complement and coagulation cascades have been upregulated, meaning angiogenesis has been promoted and the blood circulation has been relatively blocked around the tumor mass. In addition, there were small amounts of proteins involved in autoimmune defense or immune function which may have the ability to increase the risk of self-immunological defense. Moreover, some amino acid metabolisms were downregulated, leading the corresponding protein dysfunction (Figure 4(a)).

**Figure 5:**
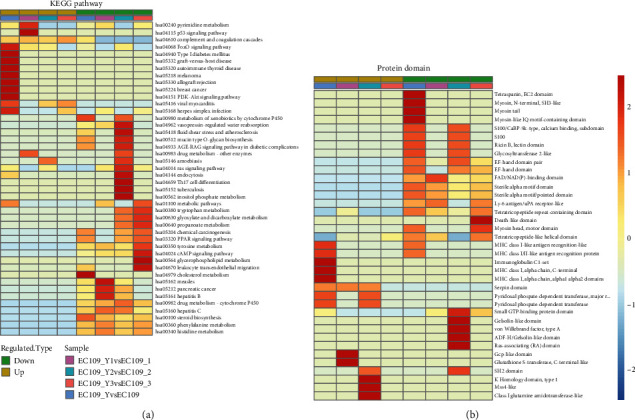
Functional clustering of the KEGG pathway (a) and protein domain (b) analysis.

Enrichment of protein structural domain analysis was applied in Figure 5(a), showing downregulation of protein belonging to the cellular matrix domain, such as myosin and myosin-like IQ motif; therefore, the cells will be more likely to collapse.  Figure 5(b) shows that protein domains were more related to cellular recognition and facilitate immune function. MHC-class I/II was an important domain which can recognize antigen-antibody before the immunological reaction happens. Moreover, proteins facilitate cellular communication and the immune function domain was upregulated, indicating inflammation would more likely happen, as the theory of carcinogenesis could be recognized as chronic inflammation which happened in cell-induced subcellular changes and may have the risk of cancer occurrence (Figure 4(b)).

### 3.5. The Functional Clustering for Association between Specific Protein and Esophageal Cancer

In addition, we conducted a clustering analysis to find the correlation between the functions of differentially expressed proteins among the comparison groups by the hierarchical clustering method. Humoral immune response (leukocyte, lymphocyte, and adaptive immune response) and regulation of receptor binding have been slightly upregulated in the biological process (*p* < 0.05). For cellular components, the MHC protein complex/MHC-1 protein complex has been upregulated in the control group meaning that T-cell immunity has been upregulated. The muscle-related cell components (myosin and myosin filament) have been significantly downregulated (*p* < 0.05), which would increase the risk of cellular scaffold collapsing. The specific peptide or structure protein binding function was significantly upregulated (including mRNA-3-UTR, peptide antigen, beta-2-microglobulin, and heparin). Moreover, the muscle-related binding activities such as structural constituent and actin filament were significantly downregulated with *p* < 0.01 (Figure 5(a)).

The FoxO pathway has been abnormally regulated which played a role in cell apoptosis, stress response, glucose metabolism, and posttranslational methylation. It may involve the carcinogenic process since the glucose is the primary energy for both normal cells and tumor cells and change the signaling pathways as well. Moreover, the autoimmune pathway which enhances cell immune function and the PI3K/Akt signaling pathway were both upregulated significantly serving as the key regulator in cellular survival and energy metabolism. The cholesterol metabolism pathway has been significantly downregulated, meaning the metabolic pathway for energy production shifted from lipid to glucose (*p* < 0.05), Protein-protein interaction network.

All differentially expressed protein database accession or sequence were compared against the STRING for protein-protein interaction. As shown in Figure 5(b), tetraspanin, myosin, and S-100 domains are in the top protein structure of differentially expressed protein, suggesting the association between specific protein and esophageal cancer.

## 4. Discussion

Esophageal carcinoma (EC) affects more than 450,000 worldwide in 2013, which is extremely aggressive and has a poor survival rate. The overall 5-year survival is lower than 10% worldwide. People with early diagnosis and locally advanced disease have better disease prognosis. The top five risk factors of EC are smoking, alcohol consumption, injury to the esophagus, chronic esophageal irritation, and obesity [21]. EC ranks fourth in China and occurs at a rate of 20-30 times than in the United States. The incidence in males is higher than females, aged over 40 years old. It is the second cancer from gastrointestinal cancers, only less than gastric cancer. Although endoscopic biopsy is a gold standard for diagnosis, however, it does not benefit patients in the early stage. Since early diagnosis is associated with disease outcomes, it is critical to find novel biomarkers with high sensitivity and specificity for detecting EC in the early stage. With advanced technology in biochemical, genetic, and imaging, the early screening and diagnostic method are critical. In this project, we performed a quantitative proteomic analysis on four comparable groups of EC samples using TMT labeling and LC-MS/MS to quantify the changes in the whole proteome of EC patients, followed by comprehensive bioinformatics analysis. The bioinformatics analysis showed that most of the differentially expressed proteins were involved in maintaining cell structure cytoskeletal integrity, intracellular trafficking, secretion, vesicular transport and signal transduction, metabolic process, stress-response, and single organism processes such as renal system and urogenital system development.

The cellular ability to prevent oxidative damage was decreased; superoxidized substances accumulate inside the cell, leading to cell apoptosis or death. Furthermore, we also found that a large amount of proteins participated in ion and inorganic hemostasis and immune regulation (adaptive immune response and lymphocyte-mediated immunity). Some cellular membrane proteins were identified; the ECM-receptor interaction pathway can influence tissue/organ morphogenesis [22]. ECM contains a large number of molecules, such as collagen, elastin, microfilaria proteins, and proteoglycans which can be involved in multiple pathways. ECM molecules also play a critical role in regulating cell adhesion, migration, differentiation, proliferation, and apoptosis [23]. The angiogenesis process is an important pathway in oncogenesis. In this study, we identified proteins regulating cytokines in the angiogenesis process, response to promoting and initiating the inflammatory process. This study results should be validated by an immunochemistry exam, such as ELISA to confirm if the protein is present in the EC cells.

## 5. Conclusion

This study applied TMT labeling proteomics followed by mass spectrometric analysis to study the EC cellular proteomic profile and identified proteins involving in oncogenesis-related pathways. TMT labeling proteomics is a new approach to engage cancer diagnosis and therapy, such as the identified protein could be used in biomarker designing, as well as the target drug therapy. Although the results of this study were promising, further study regarding sensitivity and specificity should be validated as well. Large cohort studies are also needed to evaluate the clinical significance.

## Data Availability

The data of this study are available from the corresponding author upon request.

## Supplementary Materials

Supplementary Materials Supplementary Table 1: the identified differentially expressed proteins and the annotation information for each 299 related protein and genes.
